# Role of full-thickness skin grafts in post-maxillectomy rehabilitation

**DOI:** 10.6026/973206300211221

**Published:** 2025-05-31

**Authors:** Ajay Kumar Kushwaha, Himanshu Ajay Kumar, Aftab Hossain, Anju Jha, Harsimran Singh, Sameer Gupta

**Affiliations:** 1 Department of Dentistry, Autonomous State Medical College, Kanpur Dehat, Uttar Pradesh, India; 2 Department of Oral & Maxillofacial Surgery, Maharishi Markandeshwar College of Dental Sciences & Research, (Deemed to be University), Mullana-Ambala, Haryana, India; 3 Department of Trauma Surgery and Critical Care, All India Institute of Medical Sciences, Jodhpur, Rajasthan, India; 4 Department of Pediatric & Preventive Dentistry, Patna Dental College, Patna, Bihar, India; 5 Department of Dental Surgery, Rayat Bahra Dental College and Hospital, Mohali, Punjab, India; 6 Department of Oral & Maxillofacial Surgery, Inderprastha Dental College and Hospital, Sahibabad, Ghaziabad, Uttar Pradesh, India

**Keywords:** Full-thickness skin graft (FTSG), maxillectomy, mid face defects, facial reconstruction, soft tissue reconstruction, maxillofacial defect

## Abstract

The importance of full thickness skin graft for reconstruction or Rehabilitation after Maxillectomy and mid face defect is well known. In this study we included 50 patients who underwent maxillectomy due to cancer of the mid-face and had full-thickness skin graft reconstruction. Based on the clinical examination and the subjective assessment of patients, a positive effect of the use of free skin grafts on their convalescence and rehabilitation was found. In particular, they contributed to the reduction in postoperative pain and pain associated with prosthetic stages. Full-thickness skin grafts in combination with individual prosthetic restorations are a good alternative to rehabilitation in patients who do not qualify for microsurgical treatment.

## Background:

Maxillectomy and midface defects present significant reconstructive challenges in oral and maxillofacial surgery. These defects can lead to severe functional and aesthetic impairments, affecting speech, mastication and overall quality of life. The complexity of reconstruction requires innovative solutions to restore both form and function effectively [1]. Full-thickness skin grafts (FTSGs) have emerged as a breakthrough solution in reconstructive surgery, providing superior structural and aesthetic benefits compared to other grafting techniques [2]. Their application not only enhances healing but also significantly improves patient rehabilitation outcomes. This article explores the significance of FTSGs in post-maxillectomy rehabilitation, highlighting their advantages and impact on patient recovery [3]. Loss of the maxilla and midfacial structures after tumour removal has substantial functional and aesthetic consequences [4]. Maxillectomy is a key surgical procedure for managing malignant tumors of the midface and maxilla, often requiring careful evaluation of tumor extent and surrounding anatomical structures. According to Shah and Patel, achieving clear surgical margins is essential for reducing recurrence risk and improving prognosis. Postoperative reconstruction focuses on restoring both aesthetics and function, particularly speech and swallowing. A multidisciplinary approach is crucial to ensure comprehensive treatment and effective rehabilitation [5]. Efforts have been made to classify these midfacial defects and provide appropriate algorithms for optimum reconstruction. The wide variety of free flaps that contain both soft tissue and bone offer unique properties that could be applicable depends on the defect [6]. Combinations of free tissue transfer, local flaps and maxillofacial prostheses might achieve a more ideal result than one technique alone.

Advances in osseointegration have also enhanced the ability to achieve the best function and form. No one flap or technique is sufficient to reconstruct midface defects in all patients. The choices should be tailored to the bony and soft-tissue needs of each specific defect, denture-bearing potential of the original tissues and available prosthodontic support. Use of a multidisciplinary approach to reconstruct these defects can yield excellent results [7]. The complexity of the techniques should match the desired goals and needs of each individual patient. Midface reconstruction and prosthetic rehabilitation are essential components of head and neck cancer care [8, 9]. Eskander *et al.* highlight the importance of individualized reconstructive strategies, including the use of advanced flap techniques like the scapular tip flap for optimal functional and aesthetic outcomes. Rosen *et al.* emphasize the role of maxillofacial prosthetics, particularly for hard palate and nasal defects, while noting cost as a significant barrier to access [8, 9]. Patients undergoing maxillectomy often experience extensive soft and hard tissue loss [10, 11]. Improve the integration of obturators and other prosthetic devices, thereby restoring oral function [10]. Minimizing Scarring and Contracture Ensuring better long-term stability and reducing the need for secondary interventions [11]. Further, donor site morbidity for choosing appropriate donor sites to minimize functional impairment is needed [12]. Therefore, it is of interest to describe the role of full-thickness skin grafts in post-maxillectomy rehabilitation.

## Materials and Methods:

The study included 50 patients surgically treated for jaw tumor, from whom a group was collected, in which free skin graft from the groin area was used. The group consisted of adult patients without predilection for age and gender. The exclusion criteria allowed patients with health disorders and those using stimulants (alcohol, cigarettes). The study group consisted of 35 patients with a free skin graft from the groin area, while the control group consisted of 25 patients who did not consent or were not qualified for free skin graft because of local contraindications (dermatological skin diseases of groin area, urinary tract infections UTI, perianal diseases, such as anal fissures and haemorrhoids). All of the patients were supplied with a palatal plate in the operative period. Patients qualified for surgery had to undergo a complete diagnostic test panel to get prepared for the procedure. The surgical procedure in study group consisted of complete tumor removal with negative resection margins (the radicality of the resection was assessed by intraoperative histopathological examination) The size of the graft corresponded to the size of the recipient site, considering the excess tissue resulting from the shape of the graft (lenticular) which enabled better adaptation of tissues around the donor site. The graft was cleared of adipose tissue and perforated with a needle to increase its dimensions, flexibility and allow drainage, preventing the formation of a hematoma after its introduction into the recipient site. After being sewn into the post-resection area, the free skin graft was stabilized with a gauze dressing with iodoform and a previously made palatal plate attached with a bi cortical titanium screw to the preserved fragment of the hard palate or maxilla alveolar process. After the mobilization of the surrounding tissues, the donor site was sutured in layers. The post-resection defect was supplied with gauze dressing with iodoform and a palatal plate stabilized with a screw. After surgery, patients were fed with a feeding tube or with percutaneous endoscopic gastrostomy (PEG). During hospitalization, on the 10th day the palatal plate and gauze dressing were removed, the healing was assessed, any necrotic tissues were removed and an impression was taken under the obturator. The rate of flap healing in the study group was classified into 2 groups: correct, partial; abnormal, complicated by inflammation. After the post-resection area had fully healed, the patients were qualified for adjuvant radiotherapy: 29 (78%) in study and 13 (86%) in control group. Final prosthetic restoration was performed after full recovery (retention of appliances was obtained by using tooth clamps, dental, zygomatic or titanium individual implants).

## Results:

The study group included 20 men and 15 women (M: F = 1.05), with an average age of 60.65 years (the youngest patient was 35 years old, the oldest was 75 years old). In the studied cases, the tumors were at the stage of T1-T4 (average tumor size 4 cm^2^) and the most common histopathological diagnosis was Squamous Cell Carcinoma (SCC) (51%) (Table 1). The mean stage of the lymph node criterion was N0 (43.2%) and of the organ metastases M0 (84.0%). In most cases (48%), the surgical procedure consisted of a subtotal resection of the maxilla with selective neck dissection followed by subsequent replenishment of the post-resection area with free full-thickness skin graft. The midface defects were classified in Brown's vertical classification as I and II; in horizontal as a, b, c, d. The average size of the harvested graft was 7 cm^2^. Among patients in the control group, there were 10 men and 5 women (M: F = 1.5) with an average age of 67.6 years. The most common histopathological diagnosis was also Carcinoma plano epithelial (80%) in the T1-T4 stage. The midface defects were classified in vertical and horizontal Browns' classification similarly as in study group (I, II, a, b, c, d). Full hospitalization lasted about 11 days (10.94). On average, on the 10th day (9.94) the palatal plate was removed and the first assessment of the local condition was made. Further controls of the healing took place in ambulatory care centre. In study group, proper healing of the graft was achieved in 30 (81.1%) cases, while in 7 (18.9%) there was an inflammatory complication. The average time of the full healing of the flap was 29 days (28.51) (Table 2). In the control group, a longer healing process was observed (35.1 days) with more frequent inflammatory healing complications (ulcers, purulent inflammations-28%). The result of the final histopathological examination confirmed the radicality of the procedure. The next stage of the study was the assessment of patient's rehabilitation based on medical records and telephone surveys. Based on the obtained responses in study group, pain symptoms on the 10th day after the procedure were assessed on the VAS scale at an average of 5.48 points; at the time of taking, the impression for the postoperative palatal plate 6.65 and 4 weeks after the procedure at 2.7. In the control group it was, respectively, 5.2, 7.6 and 4.6. The quality of speaking and eating, where 1-no ability to speak/eat, 10- no difficulty in speaking/ eating, was assessed, respectively, in study group at 6.8/7.9 and in control group at 5.8/6.0. The main complaint reported by patients (47%) was insufficient stability of the used obturator (Table 3).

## Discussion:

Reconstruction of post-resection midface defects is an integral part of the surgical treatment of patients with head and neck neoplasms. The development of technology has contributed to the creation of numerous methods of reconstruction aimed at obtaining the optimal aesthetic effects, postoperative comfort, but also the elimination of complications [13]. Nowadays, micro vascular grafts are considered the "gold standard" of treatment. However, in many cases this solution is not feasible due to the patient's general health condition and the degree of advancement of the disease. This fact was an incentive for the authors to seek solutions that would improve the treatment methods used so far. The results of the available studies clearly prove the effectiveness of the use of obturators in patients who have undergone maxillectomy. The main disadvantage of this solution is severe pain associated with the initial period of use. Thanks to the use of full-thickness skin grafts, this problem is eliminated, which was determined based on the conducted study [14]. Due to the large amount of tissue in this place and the invisibility of the scars, the use of the groin area as a donor site seems to be optimal. The literature describes the use of free skin grafts after resection of the maxilla: subtotal maxillectomy, total maxillectomy. In each of these procedures, a skin graft is designed to cover and protect the exposed bone surface in the tumour resection bed, facilitating wound healing [10, 11]. When controlling the healing of free skin grafts, it was not expected that it could heal completely, especially in its central part due to the lack of substrate but healing of the adjacent soft tissues was expected. In this situation, the free skin graft additionally performed the function of a dressing, which, together with the palatal plate, ensured a tight separation of the oral cavity from the nasal cavity. This resulted in optimal healing conditions for the tissues of the resection bed and a significant reduction in the pain. The most important thing is the fact that the surveyed patients improved their comfort and reduced postoperative pain, especially at the stage of making impressions in order to perform prosthetic restorations. During the further use of obturators, patients positively assessed their pronunciation and communication with the society. They also reported no significant food intake problems. The most frequent complaint was the poor stability of prosthetic restorations [9]. Davidson and Sherris seek insufficient stability of prostheses in excess tissues due to healed free skin graft; however, due to the retention mechanism of the anchoring of restorations, this conclusion was not confirmed in the analysis conducted by the authors.

According to Brown and Rogers, obturators are a useful reconstruction method in all types of maxillectomy classification. Relating to poor stability of these appliances, it seems to be very essential and challenging to optimize and improve quality of these restorations, which was the primary aim of the following study [2]. One of the possibilities to increase the stability of the obturator is implants placement around the surrounding healthy bone tissue. This enables better anchorage of the obturator and prevents micromovements and traumatization of surrounding tissues, reducing the risk of recurrence of the neoplastic process [3]. Based on the study, it can be concluded that the use of free skin flaps with an obturator is a particularly good method of treatment in patients who do not qualify for more advanced reconstructive methods, such as microvascular reconstructions. They allow for a reduction in pain in the perioperative period and reduce the risk of recurrence of the disease. The obturators used function as a temporary restoration, while the final restorations require additional anchoring in the form of implants [4]. Another important advantage of the presented methodology, using obturators, is the simplicity of the procedure presented by Brandao and Migliorati, which may be an excellent alternative to microvascular reconstructions in the time of the COVID-19 pandemic and the related logistic and personnel constraints [15]. Full-thickness skin grafts (FTSGs) play a valuable role in the rehabilitation of patients after maxillectomy and midface defects, especially when microvascular reconstruction is not feasible. They help restore oral function, improve prosthetic fitting, and support aesthetic outcomes. Although FTSGs have limitations like potential graft contraction and dependence on a well-vascularized bed, they offer a less invasive and effective option for selected patients, contributing positively to both functional recovery and psychological well-being [16].

## Conclusion:

Full-thickness skin grafts ensure optimal healing conditions and reduce postoperative pain after maxilla resection surgeries. Full-thickness skin grafts represent a major advancement in the rehabilitation of patients with maxillectomy and midface defects. Their ability to provide superior aesthetic and functional outcomes makes them a preferred choice in reconstructive oral and maxillofacial surgery. As surgical techniques continue to evolve, the role of Full-thickness skin grafts in improving patient quality of life remains indispensable.

## Figures and Tables

**Table 1:** Structure of group

**Study Group**		**Control Group**			
**Number of Patients**	**% Patients**	**Number of Patients**	**% Patients**		
Sex	Female	18	49	6	40
	Male	19	51	9	60
Age	30-50	10	27	1	6.7
	50-70	17	46	6	40
	>70	11	26	8	52.3
Type of Neoplasma	Carcinoma planoepitheliale	18	52	12	81
	Adenoid cystic carcinoma	3	10	1	6.5
	Mucoepidermoid carcinoma	3	7	0	0
	Others	10	31	2	12.3
Tumor Size	<2 cm	11	26	5	32.3
	2-4 cm	21	58	7	45.7
	>4 cm	4	13	3	21

**Table 2:** Assessment of flap's healing

**Study Group**		**Control Group**			
**Number of Patients**	**% Patients**	**Number of Patients**	**% Patients**		
Time of Full-Healing	<14	1	3	0	0
	14-28	20	57	7	45.7
	>28	13	41	8	54.3
Healing	correct, partial	30	81	-	-
	abnormal, complicated by inflammation	6	18	-	-

**Table 3:** Assessment of patient's rehabilitation

**Study Group**		**Control Group**			
**Number of Patients**	**% Patients**	**Number of Patients**	**% Patients**		
Pain in the 10th day after surgery (VAS)	<5	9	24.3	3	20
	5-8	28	75.7	12	80
	>8	0	0	0	0
Pain when taking the impression (VAS)	<5	5	13.5	0	0
	5-8	25	67.6	11	73.3
	>8	7	18.9	4	26.7
Pain after 4 weeks after surgery (VAS)	<5	34	91.9	6	40
	5-8	3	8.1	9	60
	>8	0	0	0	0
Subjective evaluation of the quality of speaking	1-4 (limited)	3	5.4	4	26.7
	5-7 (suffiecient)	24	62.1	9	60
	8-10 (correct)	11	32.4	2	13.3
Subjective evaluation of quality eating	1-4 (limited)	0	0	1	6.7
	5-7 (suffiecient)	10	29.7	11	73.7
	8-10 (correct)	26	70.3	3	20
